# An integrated transcriptome analysis in T‐cell acute lymphoblastic leukemia links DNA methylation subgroups to dysregulated *TAL1* and ANTP homeobox gene expression

**DOI:** 10.1002/cam4.1917

**Published:** 2018-12-21

**Authors:** Zahra Haider, Pär Larsson, Mattias Landfors, Linda Köhn, Kjeld Schmiegelow, Trond Flægstad, Jukka Kanerva, Mats Heyman, Magnus Hultdin, Sofie Degerman

**Affiliations:** ^1^ Department of Medical Biosciences Umeå University Umeå Sweden; ^2^ Department of Radiation Sciences Umeå University Umeå Sweden; ^3^ Department of Paediatrics and Adolescent Medicine Rigshospitalet Copenhagen Denmark; ^4^ Department of Pediatrics University of Tromsø and University Hospital of North Norway Tromsø Norway; ^5^ Children’s Hospital, Helsinki University Central Hospital, University of Helsinki Helsinki Finland; ^6^ Department of Women’s and Children’s Health Karolinska Institutet Stockholm Sweden

**Keywords:** BEX1, DNA methylation, HOXA, pediatric acute lymphoblastic leukemia, TAL1

## Abstract

Classification of pediatric T‐cell acute lymphoblastic leukemia (T‐ALL) patients into CIMP (CpG Island Methylator Phenotype) subgroups has the potential to improve current risk stratification. To investigate the biology behind these CIMP subgroups, diagnostic samples from Nordic pediatric T‐ALL patients were characterized by genome‐wide methylation arrays, followed by targeted exome sequencing, telomere length measurement, and RNA sequencing. The CIMP subgroups did not correlate significantly with variations in epigenetic regulators. However, the CIMP+ subgroup, associated with better prognosis, showed indicators of longer replicative history, including shorter telomere length (*P* = 0.015) and older epigenetic (*P* < 0.001) and mitotic age (*P* < 0.001). Moreover, the CIMP+ subgroup had significantly higher expression of ANTP homeobox oncogenes, namely *TLX3, HOXA9, HOXA10,* and *NKX2‐1*, and novel genes in T‐ALL biology including *PLCB4, PLXND1*, and *MYO18B*. The CIMP− subgroup, with worse prognosis, was associated with higher expression of *TAL1* along with frequent STIL‐TAL1 fusions (2/40 in CIMP+ vs 11/24 in CIMP−), as well as stronger expression of *BEX1*. Altogether, our findings suggest different routes for leukemogenic transformation in the T‐ALL CIMP subgroups, indicated by different replicative histories and distinct methylomic and transcriptomic profiles. These novel findings can lead to new therapeutic strategies.

## 1 INTRODUCTION

Acute lymphoblastic leukemia (ALL) accounts for 75%‐80% of all pediatric leukemia cases and is characterized by accumulation of undifferentiated blast cells in the bone marrow. Among the pediatric ALL cases, 15%‐20% are derived from the T‐cell progenitors and are classified as T‐cell ALL (T‐ALL).^1^

Recurrent molecular events associated specifically with T‐ALL have been identified, including activating mutations of NOTCH1, suppressive alterations of cell cycle regulators (9p21.3 deletions),^2^ chromosomal rearrangements involving the T‐cell receptor loci,^3^ and ectopic expression of specific transcription factor oncogenes.^4^, ^5^, ^6^ These driver oncogenes include the basic helix‐loop‐helix (bHLH) family members *TAL1* and *LYL1*; members of the HOXA and NK‐like (NKL) subclass of the ANTP homeobox gene family *TLX1*, *TLX3*, *HOXA*9*, HOXA10,* and *NKX2‐1*; and the LIM‐only domain (LMO) gene members *LMO1* and *LMO2*. However, a prognostic or therapeutic relevance of these genetic alterations has not been clearly demonstrated. Therefore, due to a lack of treatment stratifying markers, T‐ALL patients are currently only stratified based on their response to therapy, potentially overlooking important molecular prognostic information. DNA methylation alterations have been associated with prognosis in various hematological disorders.^7^, ^8^ We have previously shown, in two independent cohorts, prognostically relevant subgrouping of pediatric T‐ALL samples at diagnosis based on a CIMP (CpG island methylator phenotype) panel including 1293 gene promoter enriched CpG sites.^9^, ^10^ In both cohorts, the CIMP− subgroup, with a methylation profile closer to normal T cells, had a worse prognosis than the CIMP+ subgroup (36% vs 86% 5‐year event‐free survival in the NOPHO ALL 1992/2000 treated cohort and 29% vs 6% 3‐year cumulative incidence of relapse in the NOPHO ALL 2008 treated cohort).^9^, ^10^ The prognostic relevance was further strengthened in the NOPHO ALL 2008 treated cohort by combining CIMP status with minimal residual disease (MRD) status at the end of the induction therapy, which allowed subgrouping of high‐risk T‐ALL patients (MRD > 0.1% at day 29) (3‐year cumulative incidence of relapse in the MRD > 0.1%/CIMP− subgroup was 50% vs 12% in the MRD>0.1%/CIMP+ subgroup).^9^

The current study was aimed at investigating the biology behind the distinct T‐ALL CIMP subgroups. Integrated methylomic, genomic, and transcriptomic analysis of CIMP classified diagnostic T‐ALL samples was performed by Illumina HumMeth450K arrays, targeted exome sequencing, and RNA sequencing. The CIMP subgroups showed diverse transcriptomic profiles and different replicative histories, suggesting that the subgroups may be associated with disparate leukemogenic pathways and driver events.

## 2 MATERIALS AND METHODS

Detailed description of materials and methods is provided in the Appendix S1.

### 2.1 Patient and reference samples

All available diagnostic bone marrow or peripheral blood samples of pediatric T‐ALL patients diagnosed between years 2008‐2013 (n = 65, age < 18 years) were retrieved from the NOPHO (Nordic Society of Paediatric Haematology and Oncology) Biobank (Uppsala, Sweden). Diagnosis was based on morphology, immunophenotyping, and cytogenetic analysis, and patients were treated according to the common NOPHO ALL 2008 protocol.^11^ The regional and/or national ethics committees approved the study, and the patients and/or their guardians provided informed consent in compliance with the Declaration of Helsinki.

Publicly available methylation and gene expression data used for validation and as reference samples are listed in Table S1.

### 2.2 Methylation array analysis

The methylation data for 65 T‐ALL samples and three remission samples used in this study were generated using Human Methylation 450 K BeadChip arrays (Illumina, San Diego, CA, US) and have been deposited in the NCBI Gene Expression Omnibus (GEO) database (accession no. GSE69954; Table S1).^9^ The preprocessing, normalizing, and filtering of the data, as well as differential methylation and copy number variation analysis, are described in the Appendix S1.^9^

### 2.3 CIMP classification, epigenetic (DNAm) age, and mitotic age estimation

The T‐ALL patients were previously CIMP classified using the 1293 CpG site CIMP panel.^9^ CIMP status is based on the percentage of methylated CpG sites (average *β* value >0.4) in the panel. Samples with more than 40% methylated CpG sites in the panel were classified as CIMP+, whereas samples having less than 40% methylated CpG sites were classified as CIMP−.^9^ The previously defined cutoff^9^ for CIMP status classification at 40% methylated CpGs sites within the CIMP panel was originally set in a separate T‐ALL cohort to reflect hierarchical sample clusters,^10^ with the most divergent prognosis.^9^

DNA methylation‐based models were used to predict epigenetic DNA methylation (DNAm) age^12^ and mitotic age^13^ of the 65 diagnostic T‐ALL samples, healthy children (n = 78) (GSE36064),^14^ and sorted CD3+ T cells and CD34+ cells (GSE49618).^15^

### 2.4 Telomere length measurement

Relative telomere length (RTL) was measured by the quantitative‐PCR method described previously,^16^ with minor modifications.^17^ Details of the method are described in the Appendix S1.

### 2.5 RNA‐sequencing analysis

RNA sequencing was performed at the Science for Life Laboratory, Uppsala, Sweden, for 30 T‐ALL samples with available RNA. Sequencing libraries were constructed from a minimum of 600 ng RNA using the TruSeq Stranded Total RNA kit with Ribo‐Zero Gold treatment (Illumina). For each sample, paired‐end, strand‐specific reads with length of 125 base pairs (bp) were generated on a HiSeq2500 (Illumina) instrument. Alignment, mapping, and downstream analysis including differential gene expression and fusion detection are described in the Appendix S1.

### 2.6 Fusion transcript verification by polymerase chain reaction (PCR)

The STIL‐TAL1 fusions were confirmed by polymerase chain reaction (PCR) amplification of 64 T‐ALL samples with available DNA, using previously described primers for the most common *TAL1* breakpoint region (taldb1).^18^ One of the samples was further analyzed using primers specific for an uncommon *TAL1* breakpoint (taldb7).^19^ The PCRs included 50 ng DNA, 1X PCR Buffer II (Thermo Fisher Scientific, Waltham, MA), 0.2 mmol/L dNTP, 1.5 mmol/L MgCl2, 0.2 μmol/L primers (Eurofins, Ebersberg, Germany), and 1 unit of AmpliTaq Gold (Thermo Fisher Scientific).

### 2.7 Targeted exome sequencing

The 65 diagnostic T‐ALL and three remission samples were screened for variations in epigenetic‐associated genes (Table S2) using Haloplex Target Enrichment System (Agilent Technologies, Santa Clara, CA), and the detailed method for variant calling is described in the Appendix S1.

### 2.8 Statistical analysis

Statistical analysis was performed using SPSS v. 24 (SPSS Inc, Chicago, IL), the statistical package R v.3.4.0 (R Core Team), and SIMCA v.14.0 (Umetrics, Umeå, Sweden). All statistical tests for two sample hypotheses were two‐sided and considered significant if the *P*‐value (*P*) was <0.05. A full description of the statistical tests used is presented in the Appendix S1.

The gene set enrichment analysis (GSEA v.3.0)^20^, ^21^ of differentially expressed genes used the 13 gene cluster signatures obtained from Soulier et al.^6^

## 3 RESULTS

### 3.1 DNA methylation analysis defines distinct epigenetic T‐ALL subgroups

**Figure 1:**
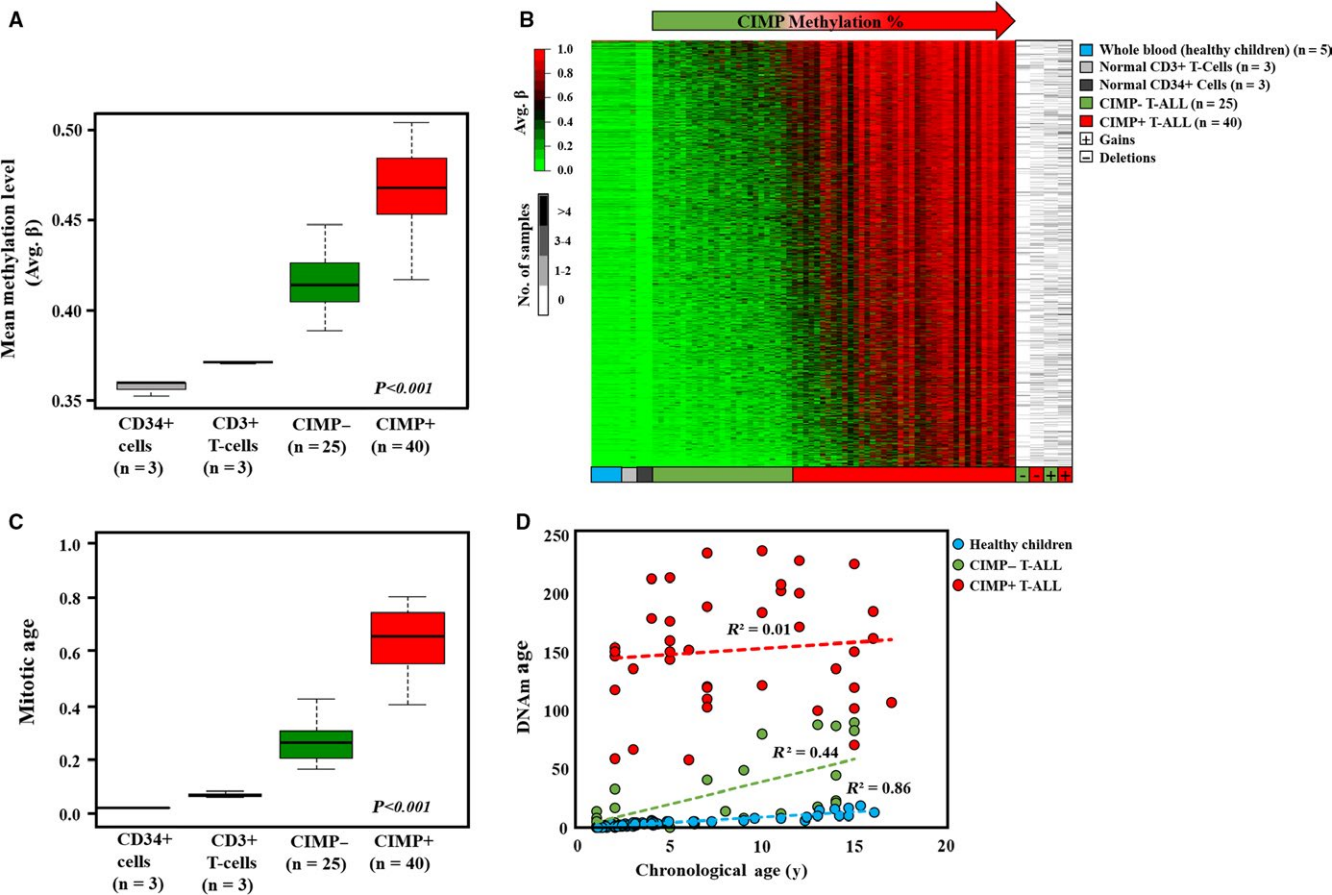
Differential DNA methylation patterns within pediatric T‐ALL. (A) Mean methylation levels (average *β*‐values) of CpGs in promoter regions (0‐1500 bp) upstream of transcription start sites of all genes in the HumMeth450k array (n = 19 298) were compared between the T‐ALL CIMP subgroups, normal sorted CD34+ and CD3+ cells using one‐way ANOVA test. (B) The heatmap (to the left) shows the average *β*‐values of 12 063 differentially methylated CpG (DM‐CpG) sites (delta *β* > 0.4 or <−0.4) between CIMP subgroups, with each CpG site shown as individual rows. The 65 T‐ALL samples, as the columns, are sorted according to increasing CIMP methylation (range 9%‐98%) along with sorted CD3+ T cells,^15^ CD34+ cells,^15^ and five whole blood samples from healthy children.^14^ The heatmap to the right shows the number of samples that have deletions or gains in the corresponding DM‐CpG region. The color intensity represents the number of samples, ranging from white (no samples) to black (>6 samples), with copy number variations. (C) Predicted mitotic age (calculated according to Yang, *et al* 2016) in normal CD3+ T cells, CD34+ cells, and CIMP T‐ALL subgroups is compared (one‐way Welch's ANOVA test). (D) Predicted DNAm age (estimated according to Horvath, 2013) of CIMP subgroups (n = 25 CIMP− and n = 40 CIMP+ samples) and healthy children (n = 78, age range 1‐16 y) is correlated with chronological age. The Pearson correlation coefficient (*R* ^2^) is given for each group

Among the 65 diagnostic T‐ALL samples in the study, 25 were classified as CIMP− and 40 were classified as CIMP+ (Table 1). The promoter methylation levels at CpG sites, up to 1500 bp upstream of the transcription start sites (TSSs) of all genes represented on the HumMeth450K array (n = 19 298) after filtering, were investigated in T‐ALL and reference samples (Figure 1A, Table 1). Both T‐ALL subgroups had higher mean promoter methylation than the normal sorted CD34+ and CD3+ T cells, and the CIMP+ subgroup showed significantly (*P* < 0.001) higher mean promoter methylation levels (0.47 ± 0.02) than the CIMP− subgroup (0.41 ± 0.01) (Table 1; Figure 1A). Differential methylation analysis revealed 12 063 differentially methylated CpG sites (DM‐CpG) in 2254 genes between the CIMP subgroups (Figure 1B). The inclusion of normal sorted immature CD34+ cells, mature CD3+ T cells, and five whole blood samples of healthy children in the heatmap showed that the DM‐CpG sites were dominated by de novo‐methylated CpG sites in the CIMP+ subgroup. Furthermore, the CIMP− samples exhibited methylation profiles more similar to normal cells (Figure 1B), irrespective of cell differentiation stage. The methylation levels of the DM‐CpGs were not associated with copy number variations as the average beta of the DM‐CpGs did not differ substantially between regions with gains or deletions (Figure 1B; Figure S1).

**Table 1:** Characteristics of the 65 CIMP classified pediatric T‐ALL samples

	CIMP−	CIMP+	*P* value
Number of samples	25	40	
Mean promoter methylation level at TSS of all genes (mean, standard deviation)	0.41 (±0.01)	0.47 (±0.02)	<0.001^a^
No. of hypermethylated CpG sites (mean, standard deviation)	19 557 (±5992)	49 692 (±11 364)	<0.001^b^
No. of hypomethylated CpG sites (mean, standard deviation)	5160 (±2013)	3709 (±1772)	0.003^a^
Enrichment of hypermethylated CpGs in different genomic regions (median, standard deviation)			
TSS1500	1.11 (±0.04)	1.12 (±0.05)	ns^c^
TSS200	0.98 (±0.09)	1.15 (±0.06)	<0.001^c^
5’UTR	1.02 (±0.06)	1.11 (±0.05)	<0.001^c^
1st Exon	1.28 (±0.19)	1.58 (±0.08)	<0.001^c^
Gene Body	0.89 (±0.03)	0.83 (±0.03)	<0.001^c^
3’UTR	0.65 (±0.08)	0.50 (±0.03)	<0.001^c^
Intergenic	1.10 (±0.04)	1.05 (±0.04)	<0.001^c^
Enrichment of hypermethylated CpGs in different CpG island regions (median, standard deviation)			
Island	1.32 (±0.19)	1.77 (±0.11)	<0.001^c^
Shelf	0.42 (±0.07)	0.27 (±0.04)	<0.001^c^
Shore	1.30 (±0.1)	1.19 (±0.06)	<0.001^c^
Open Sea	0.66 (±0.1)	0.37 (±0.06)	<0.001^c^
Chronological age/years (mean, standard deviation)	7.7 (±5.4)	8.6 (±4.8)	ns^b^
Mitotic age^d^ (mean, standard deviation)	0.27 (±0.07)	0.64 (±0.11)	<0.001^b^
DNAm age^e^(median, standard deviation)	17.8 (±31.3)	152.8 (±49.3)	<0.001^c^
Relative telomere length (median, standard deviation)	1.13 (±0.77)	0.85 (±0.46)	0.015^c^

ns, not significant (*P* value >0.05); TSS, transcription start sites

a Independent samples *t* test (equal variances assumed),

b Independent samples *t* test (unequal variances assumed),

c Mann‐Whitney *U* test.

d According to Yang et al 2016.

e According to Horvath 2013.

Using normal sorted CD34+ cells as a reference, the number of hyper‐ and hypomethylated CpG sites were calculated for each T‐ALL sample (Table 1). There was a strong correlation between the total number of hypermethylated CpG sites in the array and the percentage of methylated CpGs within the CIMP panel (*R* ^2 ^= 0.91, *P* < 0.001) (Table 1; Figure S2A). In contrast, the number of hypomethylated CpG sites correlated weakly with CIMP status (*R* ^2 ^= 0.11, *P* = 0.007) (Table 1; Figure S2B).

**Figure 2:**
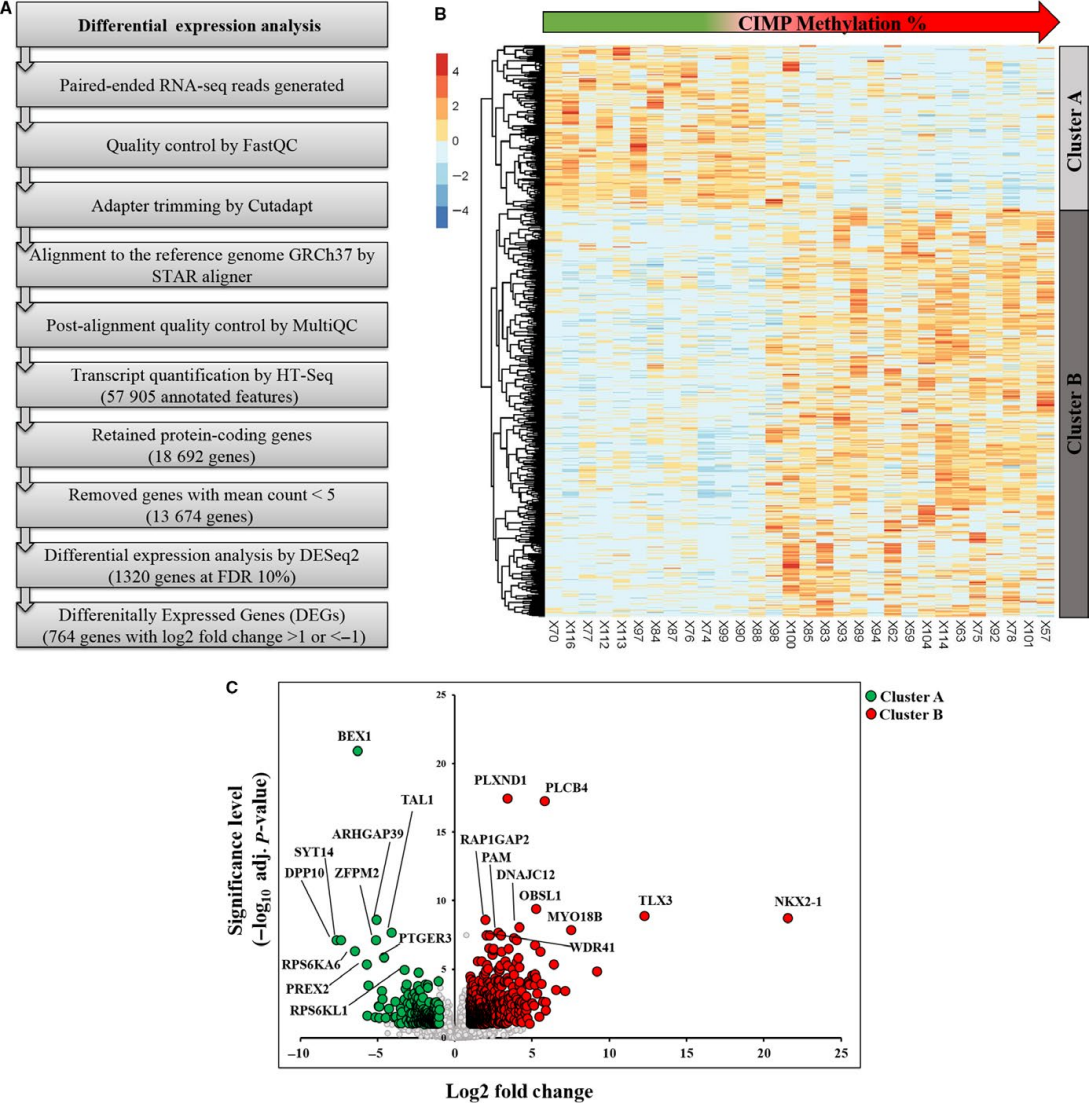
Differential transcriptomic analysis of CIMP T‐ALL subgroups. (A) The pipeline for identifying 764 differentially expressed genes (DEGs) between CIMP− (n = 12) and CIMP+ (n = 18) T‐ALL samples. Log2 fold change (LFC) was calculated using the CIMP− subgroup as reference. (B) Heatmap showing Min‐Max scaled *regularized* log transformed (rlog) counts of the 764 DEGs (rows). The samples (columns) are sorted by increasing CIMP methylation (range 11% to 98%). Unsupervised euclidean clustering separated the DEGs in two clusters. The cluster A genes (n = 216) had higher expression in CIMP− (LFC <−1) whereas the cluster B genes (n = 548) had a higher expression in CIMP+ (LFC >1) samples. (C) Volcano plot of the differential transcriptomic analysis is shown, with the top ten significant DEGs in each cluster, marked, and labeled

The hypermethylated CpG sites were enriched in CpG islands and promoter regions for both CIMP subgroups. However, CIMP+ samples displayed a significantly higher proportion of hypermethylated CpGs in these regions compared to the CIMP− samples, whereas the CIMP− samples were more frequently hypermethylated outside CpG islands and in gene body regions (Table 1; Figure S2C,D).

### 3.2 Differential replicative history of CIMP subgroups

Accumulated DNA methylation alterations are known to be associated with cell proliferation.^22^, ^23^ The proliferative history of T‐ALL samples, as well as control samples, was investigated using DNA methylation‐based models to predict mitotic age^13^ and epigenetic DNA methylation (DNAm) age,^12^ which were then correlated with the patients’ chronological age and CIMP status. As expected, the predicted mitotic age was higher in the leukemic T‐ALL samples than the sorted CD3+ T cells and CD34+ cells (Figure 1C). However, the CIMP+ subgroup had a significantly older mitotic age than the CIMP− subgroup (0.64 ± 0.11 vs 0.27 ± 0.07, *P* < 0.001) (Table 1; Figure 1C).

Similarly, the predicted DNAm age was higher in leukemic cells than normal healthy blood cells from children (n = 78) (Figure 1D) and the CIMP+ subgroup was estimated epigenetically older than the CIMP− subgroup (152.8 ± 49.3 years vs 17.8 ± 31.3 years, *P* < 0.001) (Table 1; Figure 1D). As in healthy children (*R* ^2 ^= 0.86, *P* < 0.001), DNAm age was correlated with chronological age in CIMP− samples (*R* ^2 ^= 0.44, *P* < 0.001), but this correlation was not seen in CIMP+ samples (*R* ^2 ^= 0.01, *P* = 0.53) (Figure 1D).

A longer proliferation history and an older epigenetic age of the CIMP+ subgroup were further supported by significantly shorter relative telomere length (RTL) than the CIMP− group (0.85 ± 0.46 in CIMP+ vs 1.13 ± 0.77 in CIMP−, *P* = 0.015) (Table 1).

### 3.3 Differential transcriptomic analysis of the CIMP subgroups

To explore the transcriptome and the subsequent functional differences between the CIMP subgroups, we performed RNA sequencing of 30 T‐ALL samples (12 CIMP− and 18 CIMP+). An average of 76 million (m) reads (range 56.7‐131.9 m) was generated with 97.9% of the reads mapping to the reference genome.

Differential gene expression analysis identified 764 significantly differentially expressed genes (DEGs) out of which 216 genes had a higher expression in CIMP− subgroup (log2 fold change (LFC) <−1) and 548 genes had a higher expression in the CIMP+ subgroup (LFC > 1) (Figure 2A‐C;Table S3). Enrichment analysis of the genes with a higher expression in the CIMP+ subgroup (cluster B) (Figure 2B) revealed the enrichment of G‐protein signaling pathways, including regulation of cyclic‐AMP (cAMP), among the top most significant pathways (Table S4). The genes with a higher expression in CIMP− subgroup (cluster A) were enriched in pathways associated with transcriptional regulation of granulocyte development and mTORC2 (mammalian target of rapamycin complex 2) signaling (Table S4).

### 3.4 Epigenetic regulators and CIMP subgroups

Mutations in specific epigenetic regulators have been associated with T‐ALL.^24^ The CIMP subgroups (65 diagnostic T‐ALL samples and three remission samples) were investigated for variations in genes involved in epigenetic regulation by targeted exome sequencing (Table S2). In addition to exome sequencing, we examined gene expression in 30 diagnostic T‐ALL samples to investigate whether the CIMP classification correlated with dysregulated epigenetic regulators.

The targeted sequencing generated an average of 1 m reads (range 0.3‐3.1 m reads), and 43 variations in 11 genes were retained after filtering (Figure S3A,B; Table S5). All identified variants were confirmed in samples analyzed by RNA sequencing by manually inspecting BAM files in IGV (except for the PHC2 gene that had no coverage). A majority of the identified variants were predicted as “benign,” and no correlation between variations in epigenetic regulators and CIMP methylation phenotype could be observed (Figure S3B; Table S5). Expression analysis showed variable expression levels of epigenetic‐associated genes within the T‐ALL samples, but no correlation with CIMP status (Figure S4) could be detected.

### 3.5 CIMP status correlated with known T‐ALL subtypes

**Figure 3:**
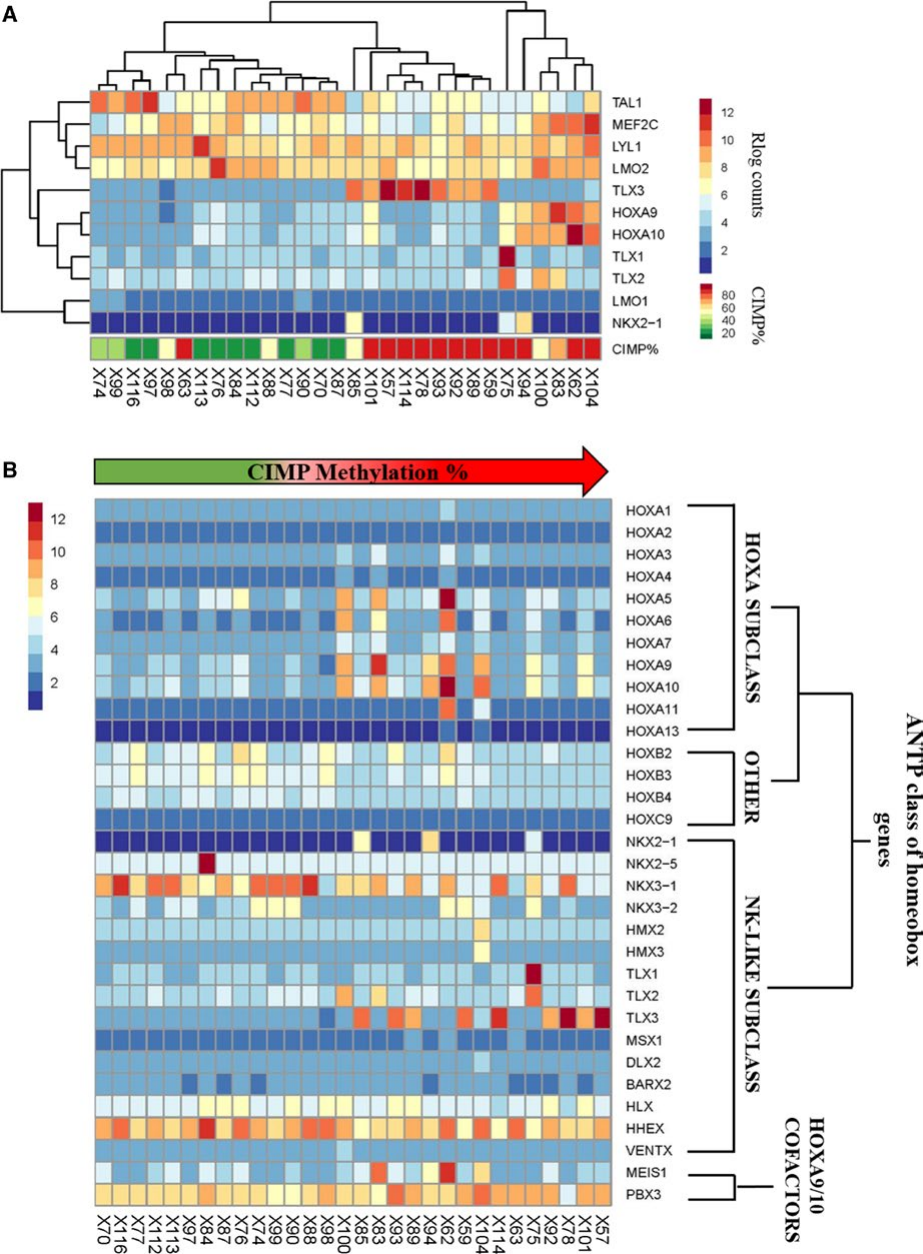
Transcriptional subtypes of T‐ALL and CIMP status. (A) The heatmap shows unsupervised clustering of 30 T‐ALL samples based on the gene expression (in rlog counts) of 11 transcription factors known to be overexpressed in T‐ALL. CIMP methylation percentage of the clustered samples is presented below the heatmap. (B) Gene expression (rlog counts) profile of the ANTP class of homeobox genes and cofactors is shown for the T‐ALL samples sorted by increasing CIMP methylation percentage (range 11%‐98%)

Transcriptomic analysis of the CIMP subgroups identified a number of known T‐ALL drivers such as *TAL1 *(LFC −4.1)*, TLX3 *(LFC 12.2), and *NKX2‐1* (LFC 21.5) among the top most significant DEGs (Figure 2C) as well as *HOXA9 *(LFC 4.6), *HOXA10 *(LFC 4.8), and *MEF2C *(LFC 2.4) implicated as differentially expressed (Table S3). The sample clustering based on the gene expression profiling of known T‐ALL drivers correlated with CIMP methylation status (Figure 3A). The *TAL1* overexpression was associated with CIMP− status, and the *HOXA9/10* as well as the *TLX1/2/3* clusters was restricted to the CIMP+ samples (Figure 3A). High *TLX3* expression was seen in 9/18 CIMP+ samples but not in CIMP− samples (0/12) (Figure 3A). Since *TLX1/2/3*, *NKX2‐1,* and *HOXA *genes belong to the same ANTP homeobox gene family,^25^ we performed a comprehensive expression analysis including all members of the gene family along with the known HOXA cofactors *MEIS1* 26 and *PBX3* 27 (Figure 3B). Specific members of the *HOXA* and *NKL* subclass had higher expression in the CIMP+ subgroup (Figure 3B; Table S3).

The association of CIMP subgroups with *TAL1* and homeobox gene expression profiles was further supported by gene set enrichment analysis (GSEA) of the identified 764 DEGs in our study with the 13 T‐ALL gene expression cluster signatures defined by Soulier et al^6^ (Table S6). Genes with a higher expression among CIMP− samples (cluster A) were significantly enriched for genes in Soulier's C2 (*P* < 0.001) and C3 (*P* < 0.001) clusters, both of which characterize *TAL1* expressing T‐ALL patients. Similarly, the genes with a higher expression in the CIMP+ samples (cluster B) correlated with the homeobox‐associated C8 (*P* = 0.02), C9 (*P* = 0.04), and C11 (*P* = 0.01) clusters (Table S6).

### 3.6 STIL‐TAL1 fusions in CIMP− subgroup

**Figure 4:**
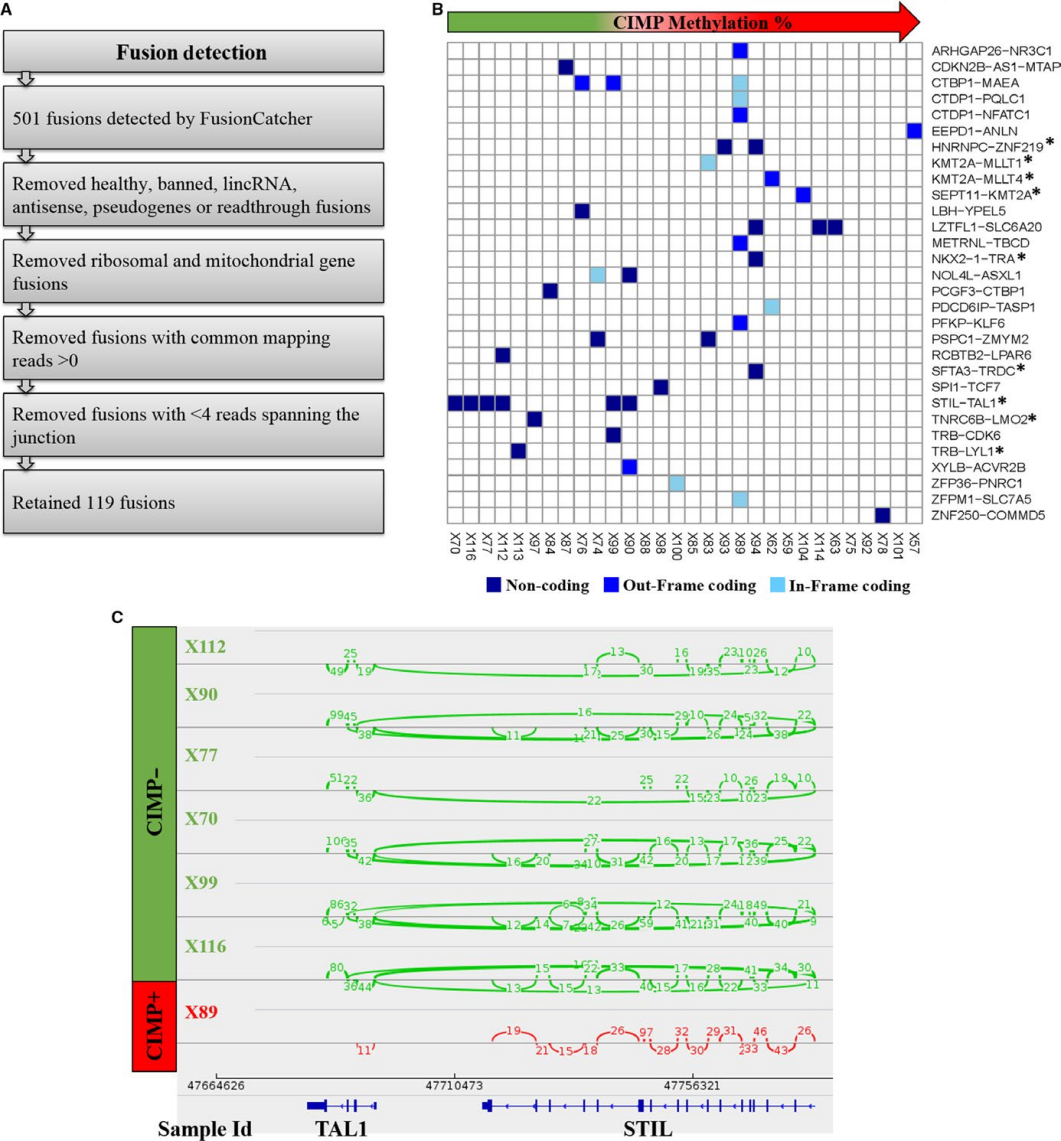
Fusion detection in pediatric T‐ALL. (A) The pipeline used for detection of fusion genes from RNA‐sequencing data by FusionCatcher is shown. (B) The heatmap shows the occurrence of identified gene fusions in relation to CIMP status in the 30 T‐ALL samples. The fusions marked with (*) are known recurrent translocations in T‐ALL. (C) Sashimi plots showing the junctions supporting the STIL‐TAL1 fusions identified in (B) in six CIMP− samples (green). A reference sample (CIMP+ sample) with no fusion detected is shown in red

The majority of oncogenes implicated in T‐ALL biology are activated by genomic alterations.^3^ We used FusionCatcher to identify translocations in the 30 T‐ALL samples that were analyzed by RNA sequencing (Figure 4A). After filtering, 119 translocations remained, represented by 30 unique gene combinations (Figure 4A,B; Table S7). We identified genes with high expression in the transcriptome analysis that was associated with the identified translocations, including NKX2‐1‐TRA, TRB‐LYL1, and most notably STIL‐TAL1 translocations (Figure 4B). Interestingly, the STIL‐TAL1 fusions were found only in the CIMP− subgroup (6/12 CIMP− and 0/18 CIMP+ samples) (Figure 4B,C).

The presence of STIL‐TAL1 translocations in the CIMP− subgroup was verified by PCR, using primers^18^ designed for the most commonly occurring TAL1 deletion breakpoint 1 (taldb1) and the STIL deletion breakpoint 1 (stildb1). STIL‐TAL1 translocations were observed in 42% (10/24) of CIMP− samples compared with 5% (2/40) of CIMP+ samples (Figure S5A). All samples but one (X70) that were positive for the fusion by RNA sequencing were verified (Figure S5A). Upon visual inspection of the alignment data using IGV, X70 was found to carry a rare TAL1 breakpoint, namely TAL1 deletion breakpoint 7 (taldb7), that was later verified by a different pair of PCR primers^19^ (Figure S5B).

### 3.7 Novel genes in T‐ALL biology

In addition to the *TAL1* and ANTP homeobox gene family members, several genes not previously associated with T‐ALL biology were identified among the top most significant DEGs between the CIMP subgroups, including *BEX1, PLXND1, PLCB4, *and *MYO18B* (Figure 2C). The brain‐expressed X‐linked 1 (*BEX1*) gene, located on the X chromosome, had the lowest adjusted *P*‐value, with a higher expression in the CIMP− subgroup (LFC‐6.3). *BEX2*, another member of the BEX gene family, was also differentially expressed (LFC‐2.2) (Table S3). Since epigenetic mechanisms regulate X chromosome inactivation in females, we analyzed whether *BEX1* or *BEX2 *expression was associated with gender of the patients. The expression of both, *BEX1* and *BEX2*, did not correlate with the gender of the patients (*P* = 0.93 and *P* = 0.53, respectively, Mann‐Whitney *U* test).

In contrast to the *BEX *genes, the *PLXND1* (Plexin D1), *PLCB4 *(Phospholipase C) genes had significantly higher expression in the CIMP+ subgroup (LFC 3.4 and LFC 5.8, respectively) (Figure 2C; Table S3). *PLXND1* has previously been associated with intra‐thymic migration of thymocytes during T‐cell development,^28^ and both, *PLXND1* and *PLCB4*, have been implicated in various cancers^29^, ^30^ but not in T‐ALL. The *MYO18B* (Myosin XVIIIB) gene, a tumor suppressor gene associated with lung,^31^ ovarian,^32^ and colorectal cancer,^33^ was strongly expressed (LFC 7.5) in a set of CIMP+ cases (7/18) (Figure 2C).

### 3.8 Validation of DEGs in a separate T‐ALL cohort and normal stimulated T cells

In order to relate the expression levels of selected DEGs in the CIMP subgroups to normal cells, we used our previously published gene expression array data^10^ of a separate cohort of pediatric T‐ALL patient samples (11 CIMP− and 6 CIMP+) and normal stimulated T cells (n = 2). Despite the limited sample size, we observed that the *TAL1, BEX1, *and *BEX2 *genes were weakly expressed in normal and CIMP+ samples but significantly upregulated in the CIMP− subgroup (Figure S6). Conversely, the *PLXND1, PLCB4, HOXA9, HOXA10, TLX3*, and *NKX2‐1* genes had higher expression in the CIMP+ subgroup, compared to the normal T cells and CIMP− leukemias (Figure S6).

### 3.9 Integrated promoter methylation and gene expression analysis for the DEGs

**Figure 5:**
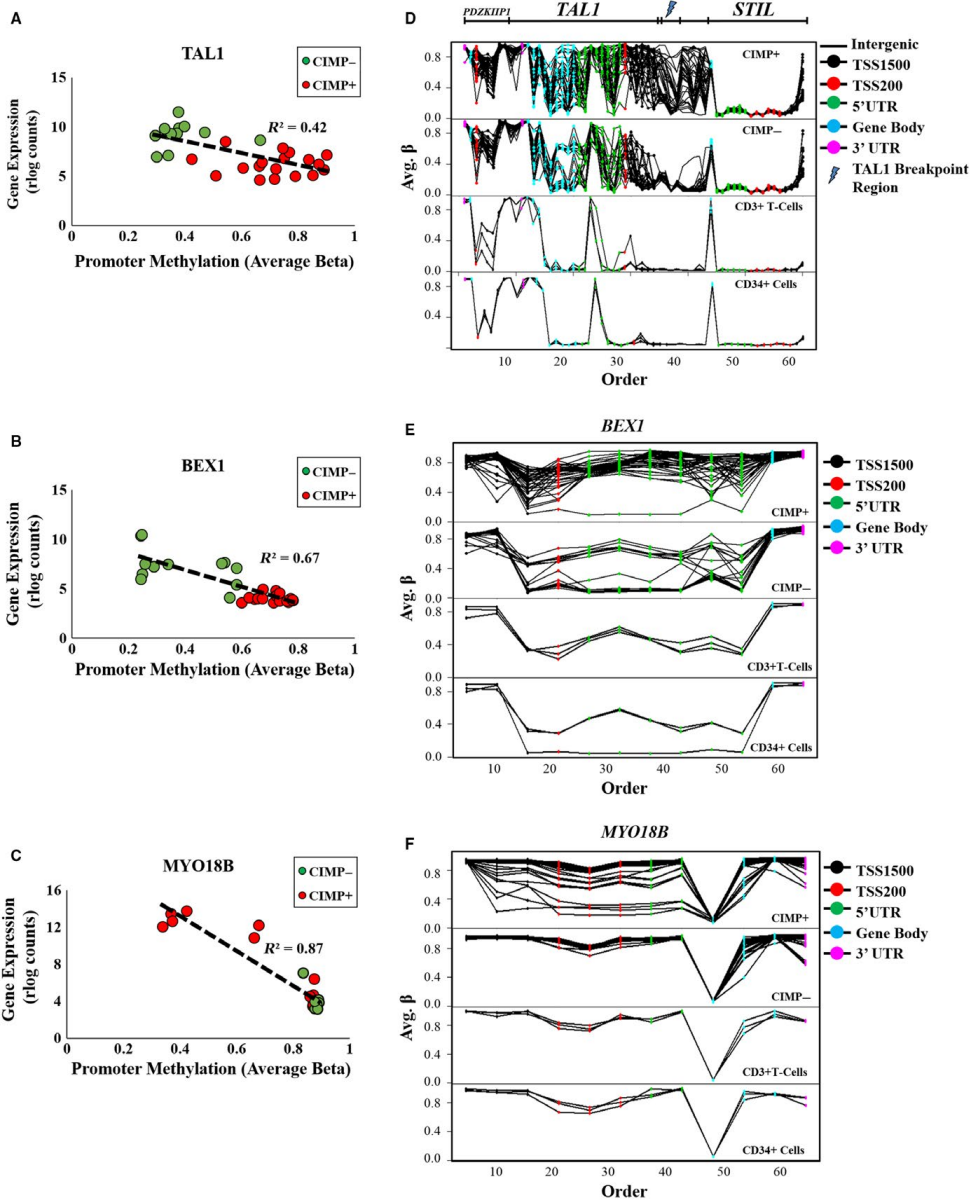
Promoter methylation of differentially expressed genes. Mean promoter methylation (TSS1500, TSS200 and 5’UTR) of (A) TAL1, (B) BEX1, and (C) MYO18B was correlated with gene expression (rlog counts) in the 30 T‐ALL samples using Pearson correlation (*R* ^2^). DNA methylation (Avg. *β* value) of CpG sites in (D) the TAL1 regulon including the neighboring STIL and PDZKIP1 genes (E) BEX1 and (F) MYO18B was plotted for the CIMP+ (n = 40), CIMP− (n = 25) and normal sorted CD3+ T cells (n = 3) and CD34+ cells (n = 3). Each CpG site is colored according to the annotated genomic region, and the TAL1 breakpoint region is marked

An integrated promoter methylation and gene expression analysis, including genes located on the X chromosome, were performed on the 30 T‐ALL samples with both transcriptomic and methylomic data. Promoter methylation data (TSS1500, TSS200, 5’UTR) were available for 746 of the 764 DEGs. A significant correlation between methylation and gene expression was observed in 281 of the DEGs, and 79% (n = 222) of these genes had negative correlations (Pearson correlation *R* range −0.36 to −0.93) (Table S3). Among the genes with the strongest negative correlation were *TAL1* (*R* ^2 ^= 0.42), *MYO18B (R^2 ^*= 0.86) and *BEX1 *(*R* ^2^ *^^= *0.67) (Figure 5A‐C; Table S3). Neither the *HOXA9/10* genes nor the *TLX3* gene expression was significantly correlated with promoter methylation (Table S3).

Methylation profiling at single CpG site resolution of the *TAL1, BEX1,* and *MYO18B* genes was performed in the CIMP− (n = 25) and CIMP+ (n = 40) samples, along with sorted CD3+ and CD34+ cells. (Figure 5D‐F). Analysis of the *TAL1* regulon, including the neighboring *PDZKIP1 *and *STIL* genes, revealed that the TSS1500 promoter region and the intergenic region between *TAL1* and its immediate 5´ neighbor *STIL* were methylated in the CIMP+ subgroup in contrast to CIMP− and reference samples (Figure 5D). This region of variable methylation between the CIMP subgroups encompassed the *TAL1*‐breakpoint region, frequently involved in translocations (Figure 5D).

The methylation level of *BEX1* was most variable in the TSS200 and 5’UTR promoter region, in which a number of CIMP− samples showed hypomethylation compared to CIMP+ and reference cells (Figure 5E).

The *MYO18B* gene promoter was methylated in the CIMP− subgroup, sorted CD3+ and CD34+ cells but was hypomethylated (TSS200 and 5’UTR region) in a few CIMP+ samples that showed increased gene expression (Figure 5C,F).

## 4 DISCUSSION

We have previously shown prognostic relevant subgrouping of pediatric T‐ALL samples at diagnosis based on DNA methylation CIMP (CpG island methylator phenotype) status. In this study, the biology behind T‐ALL DNA methylation subgroups has been investigated which was previously unknown. An integrated methylomic, genomic, and transcriptomic analysis identified links between CIMP status and known oncogenic drivers in T‐ALL, suggestive of different routes for cellular transformation in the methylation subgroups.

DNA methylation alterations are known to accumulate with increasing population doublings,^23^ and we have previously observed overlapping hypermethylation patterns between immortalized T‐cell in vitro cultures and CIMP+ T‐ALL patient samples, suggesting the association between accumulation of methylation alterations and proliferative history.^22^ In the current study, analysis of predicted mitotic and epigenetic DNAm age and telomere length analysis further support that CIMP+ cells are epigenetically older than the CIMP− cells.

Mutations and altered gene expression of DNA methyltransferases and polycomb‐associated genes have been implicated in T‐ALL biology.^24^ Although genetic variants in these genes were identified in some T‐ALL samples, an association between CIMP status and genomic or transcriptomic dysregulation of epigenetic regulators was not detected.

To further characterize the epigenetic subgroups, we performed an exploratory transcriptomic analysis of protein coding genes. We identified a considerable number of differentially expressed genes as well as enriched signaling pathways between the CIMP subgroups. Interestingly, genes with a higher expression in the CIMP− subgroup were enriched in the mTOR signaling pathway which has been shown associated with increased leukemia‐propagating potential in individual T‐ALL clones.^34^

Among the differentially expressed genes, previously known T‐ALL driver oncogenes, such as *TAL1, TLX3, HOXA9, *HOXA10, and *NKX2‐1, *were identified*. *These oncogenic transcription factors have been previously described as markers for T‐ALL subgrouping based on gene expression profiles.^4^, ^5^, ^6^, ^35^

*TAL1* is overexpressed in approximately 60% of T‐ALL cases, and among these cases, about 30% are known to exhibit this phenotype due to a ~90 kb microdeletion that translocates the *TAL1* gene with the promoter of the neighboring STIL gene.^36^ We found that the CIMP− subgroup was strongly associated with increased *TAL1* gene expression, and a higher frequency of STIL‐TAL1 fusions was observed within this group. *TAL1* overexpression may also occur as a consequence of TAL1‐TCRA/D translocations (~5% of *TAL1* expressing T‐ALL),^37^ or non‐coding microinsertions that generate super‐enhancers.^38^, ^39^ In the 30 T‐ALL samples that were RNA‐sequenced, no TAL1‐TCRA/D translocations were observed, but this could be explained by inefficient alignment to the TCR regions in the RNA‐sequencing analysis. Not all CIMP− samples with high *TAL1* expression had the STIL‐TAL1 fusion, reaffirming that *TAL1* expression can be regulated by other mechanisms than translocations. One of these mechanisms could be epigenetics as shown earlier.^40^, ^41^ A strong negative correlation between *TAL1* promoter methylation and gene expression was observed in this study, corroborating similar findings by us and others.^10^, ^42^, ^43^ Interestingly, the high‐resolution methylation analysis allowed detailed analysis of the *TAL1* regulon and showed that the variable methylated region between CIMP subgroups encompasses the *TAL1* breakpoint region for the STIL‐TAL1 fusion. The CIMP− samples showed low methylation in the breakpoint region as compared to the CIMP+ subgroup which could explain the higher frequency of STIL‐TAL1 fusions in the CIMP− subgroup. A link between low methylation and high frequency of STIL‐TAL1 translocation has been previously observed.^44^, ^45^

The CIMP+ group was overrepresented by a higher expression of homeobox genes, specifically the HOXA and NKL subclass of the ANTP gene family. The *HOXA9* and *HOXA10 *genes belong to the HOXA subclass of the ANTP family, which also includes the NK‐like subclass comprising of NKX‐ and TLX‐genes.^25^ The mechanisms leading to the overexpression of these genes in the CIMP+ could not be determined except for the translocation of NKX2‐1‐TRC found in one CIMP+ sample that overexpressed *NKX2‐1.* Gene expression of *HOXA9, HOXA10, TLX1, TLX2, TLX3, *and *NKX2‐1* did not correlate with promoter methylation, and it remains to be evaluated if the differential expression of the homeobox genes contributed to the divergent methylation profiles of the CIMP subgroups.

It has previously been shown that T‐ALL samples can be classified based on gene expression signatures driven by transcription factor oncogenes and that these signatures correlate with transcriptional profiles of different stages of thymocyte development.^5^ *TAL1 *expressing T‐ALL samples have previously been shown to correlate with the late cortical and mature stage of T‐cell development whereas homeobox gene‐driven T‐ALLs were associated with the early cortical, double‐negative stages of T‐cell development.^5^ Despite the correlation of *TAL1* and homeobox gene expression with CIMP classification, the CIMP subgroups did not correlate with the immunophenotype stage based on EGIL (European Group for the Immunological characterization of leukemias) classification.^9^ Future methylome and transcriptome analysis of sorted T cells from different stages of thymocyte development may help elucidate the relationship between CIMP subgroupings and T‐cell differentiation.

The transcriptome analysis also identified differentially expressed genes between CIMP subgroups that had not been previously linked to T‐ALL biology, including *BEX1, PLXND1*, *PLCB4, *and *MYO18B*. The *MYO18B* gene has previously been described as a tumor suppressor gene whose expression was shown to be regulated by epigenetic mechanisms in lung,^31^ ovarian,^32^ and colorectal cancers.^33^ Its relevance for hematological malignancies is largely unknown but we have shown dysregulated gene expression of *MYO18B* in pediatric T‐ALL. In contrast to lung cancer,^31^ where promoter hypermethylation of this gene in transformed cells was associated with gene silencing, we observed that promoter hypomethylation of *MYO18B* was associated with upregulation of gene expression in a set of CIMP+ T‐ALL samples. Further investigations are, however, needed to evaluate whether this gene has an oncogenic or a tumor suppressor role in T‐ALL.

The *PLXND1 *gene has been implicated in intra‐thymic migration of thymocytes during T‐cell development, is a transcriptional target of the T‐ALL‐associated NOTCH signaling pathway, and has been found to be upregulated in prostate cancer.^28^, ^29^ *PLCB4* has also been associated with various cancers such as gastrointestinal tumors^46^ and melanoma.^30^ The BEX family genes, namely *BEX1* and *BEX2, *were significantly upregulated in the CIMP− subgroup, and we showed a negative correlation of promoter DNA methylation with gene expression for both *BEX *genes in the T‐ALL samples. The expression of *BEX1* and *BEX2* has been previously shown to be regulated by epigenetic mechanisms including promoter methylation.^47^ Both *BEX1* and *BEX2* have been described as tumor suppressor genes in glioma^47^ and acute myeloid leukemia (AML).^48^, ^49^ However, the function and prognostic relevance of these genes in T‐ALL biology remain to be evaluated.

Altogether, our findings suggest the existence of different routes for leukemogenic transformation in the CIMP− and CIMP+ subgroups of T‐ALL, indicated by their distinct methylomic and transcriptomic patterns. We have previously shown that CIMP classification at diagnosis can improve risk stratification of MRD‐defined risk categories after induction therapy.^9^ Summarizing the existing findings from clinical, genetic, epigenetic, and transcriptomic analysis of the CIMP subgroups, in this and our previous studies^9^, ^10^, ^22^ reveal that CIMP− patients have a worse prognosis, with high white blood cell counts at diagnosis, younger predicted epigenetic and mitotic age, and higher *TAL1* expression. It can be extrapolated that the regulation of *TAL1*, either by promoter methylation or translocations, renders the prognosis of the CIMP− subgroup unfavorable. In a previous study, the presence of STIL‐TAL1 fusion in T‐ALL resulted in a significantly inferior overall survival as well as relapse‐free survival.^50^ Furthermore, in the same study, STIL‐TAL1+ T‐ALL had a significantly shorter time of disease onset in murine models which could explain the younger epigenetic and mitotic age as well as longer telomere length in the CIMP− subgroup. However, the impact of *TAL1* on T‐ALL prognosis is still debatable as other studies report better outcome for *TAL1* expressing T‐ALL.^51^ The higher expression of mTOR signaling pathway in CIMP− subgroup can also be speculated to contribute to the worse prognosis of this particular group since previous studies have shown the association of activated mTOR pathway with poor clinical outcome,^52^, ^53^ owing to the role of PI3K/Akt/mTOR pathway in the survival of drug‐resistant leukemia‐initiating cells.^54^

On the other hand, the CIMP+ subgroup have a better prognosis, are epigenetically and mitotically older, with hypermethylation in promoter regions of polycomb target genes, and have a higher expression of homeobox genes. Especially for CIMP+ classified patients, demethylating therapeutic agents, such as decitabine and azacitidine, have the potential to be included in ALL treatment protocols. Decitabine was well tolerated in a clinical trial phase 1 study in 39 relapse ALL patients.^55^

Recently, it was also shown that classification based on gene mutations (*NOTCH1, FBXW7, PTEN,* and *Ras)* combined with MRD and WBC status improves risk stratification of pediatric T‐ALL patients.^56^ The next step will be to combine the mutational classification with CIMP subgrouping in larger cohorts, to evaluate the interplay of these prognostic biomarkers and their individual and combined potential to improve therapy stratification of T‐ALL. Functional analysis of the novel genes in T‐ALL biology identified in this study (*BEX1, PLXND1*, *PLCB4, *and *MYO18B)* will further evaluate their role in T‐ALL pathogenesis and therapy response.

## CONFLICT OF INTEREST

The authors declare no competing interests.
