# Supplementary figures and images for: An integrated transcriptome analysis in T‐cell acute lymphoblastic leukemia links DNA methylation subgroups to dysregulated TAL1 and ANTP homeobox gene expression
Source: Cancer Med. 2018 Dec 21;8(1):311–24. doi: 10.1002/cam4.1917 (PMC6346238; doi:10.1002/cam4.1917)

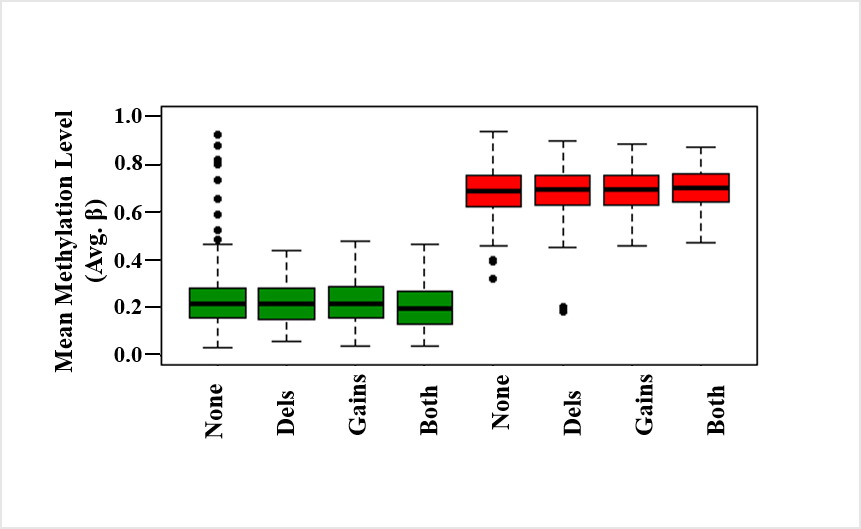

Supplement: Supplementary file 1 [file CAM4-8-311-s001.tif]

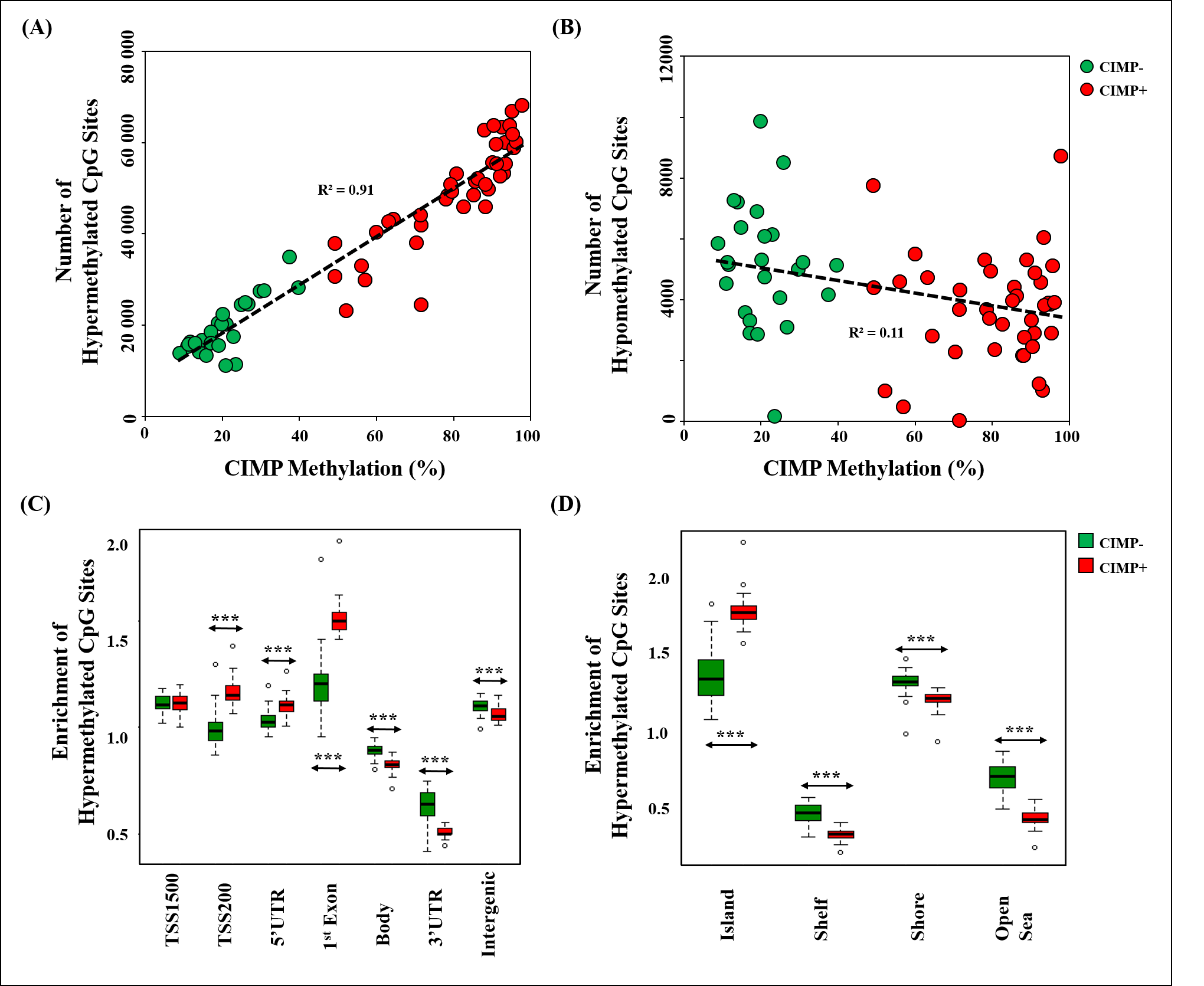

Supplement: Supplementary file 2 [file CAM4-8-311-s002.tif]

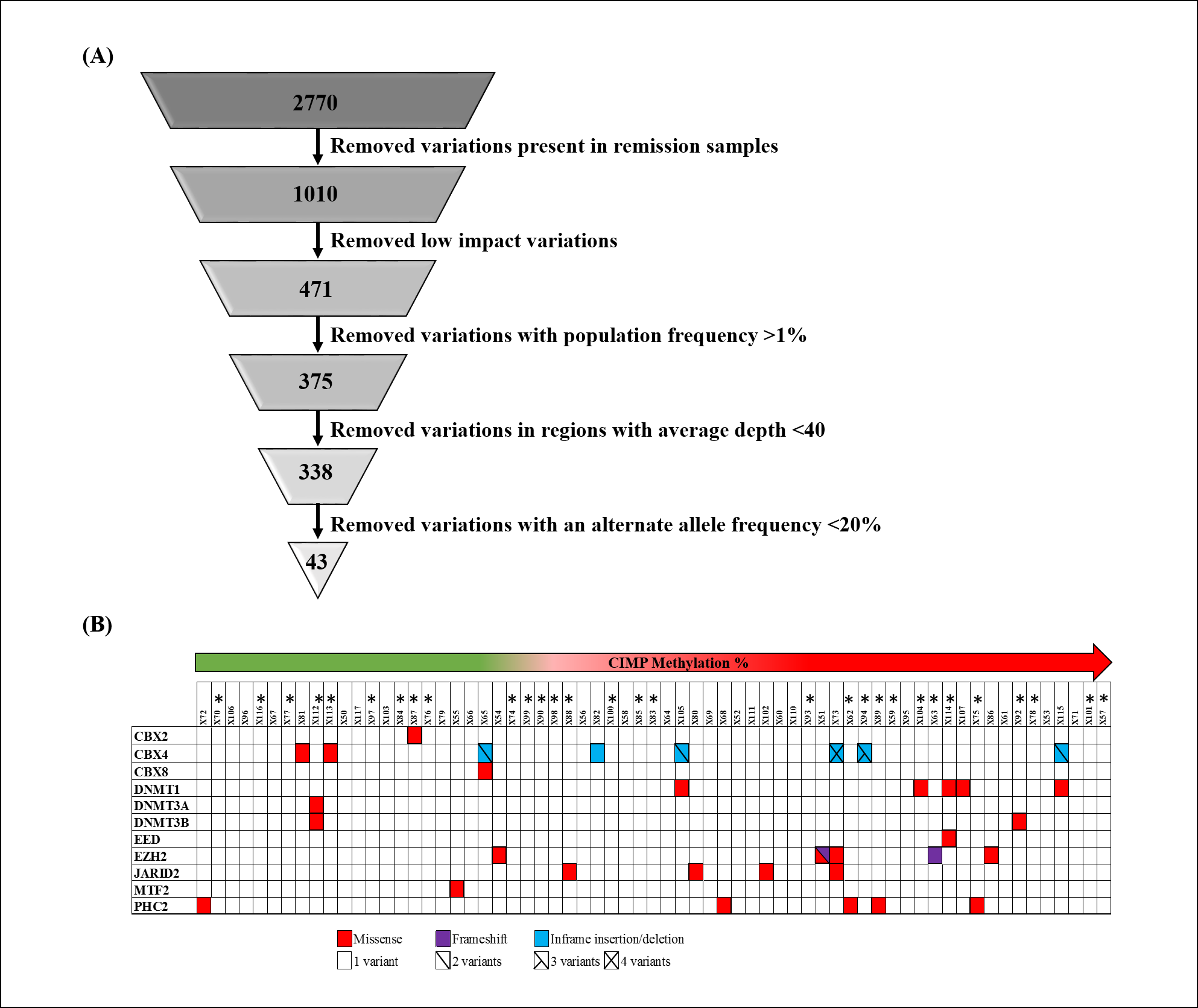

Supplement: Supplementary file 3 [file CAM4-8-311-s003.tif]

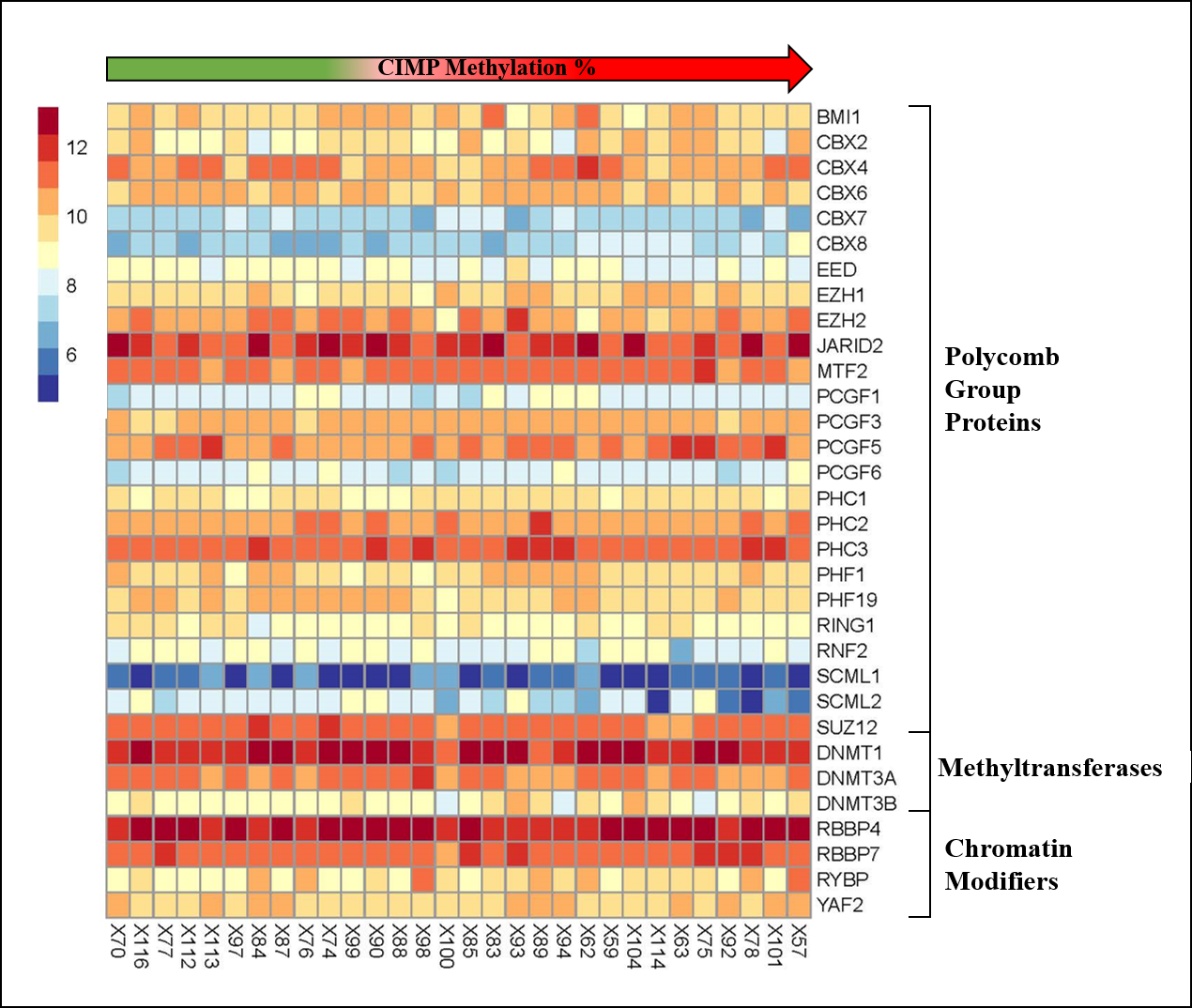

Supplement: Supplementary file 4 [file CAM4-8-311-s004.tif]

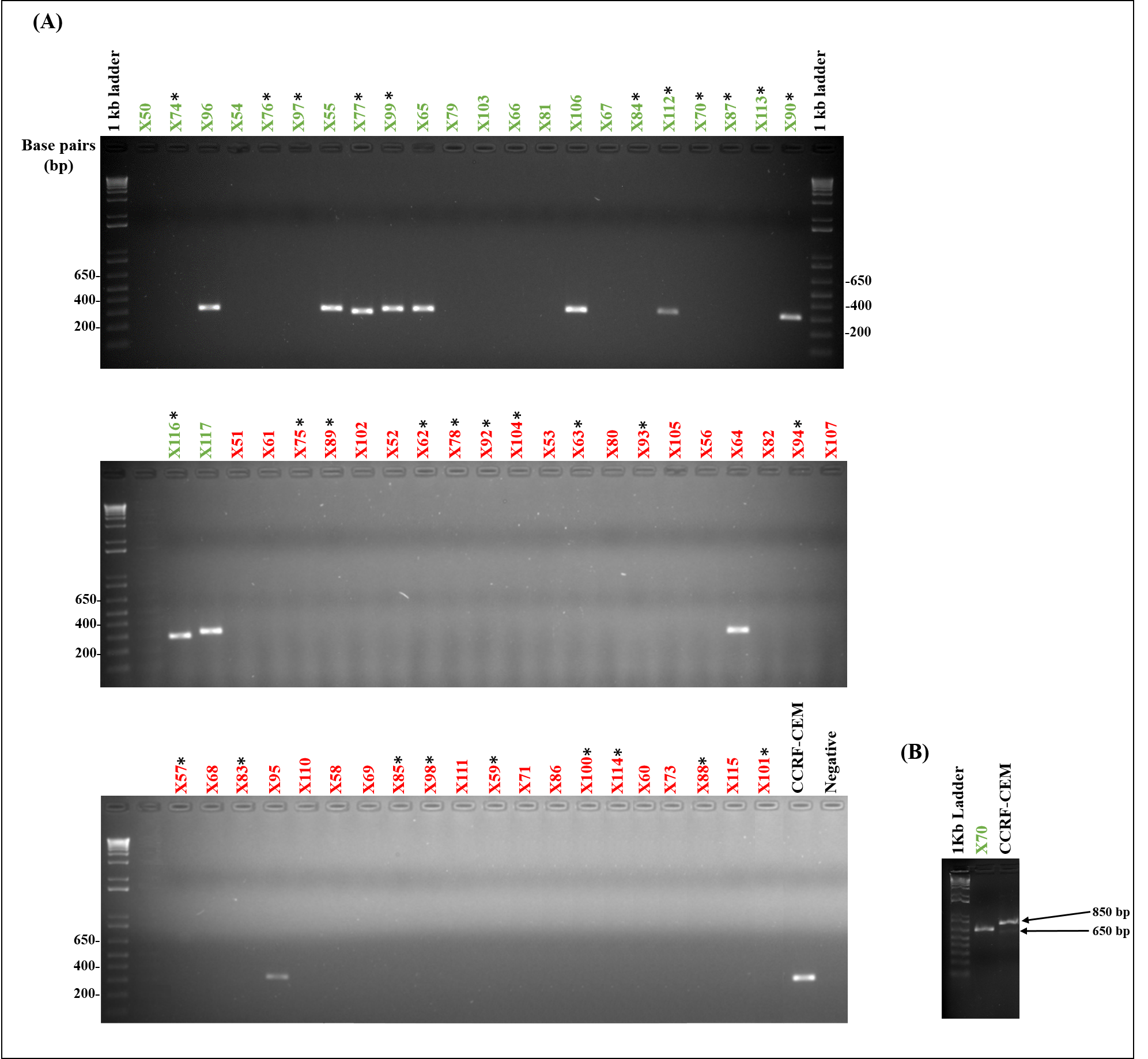

Supplement: Supplementary file 5 [file CAM4-8-311-s005.tif]

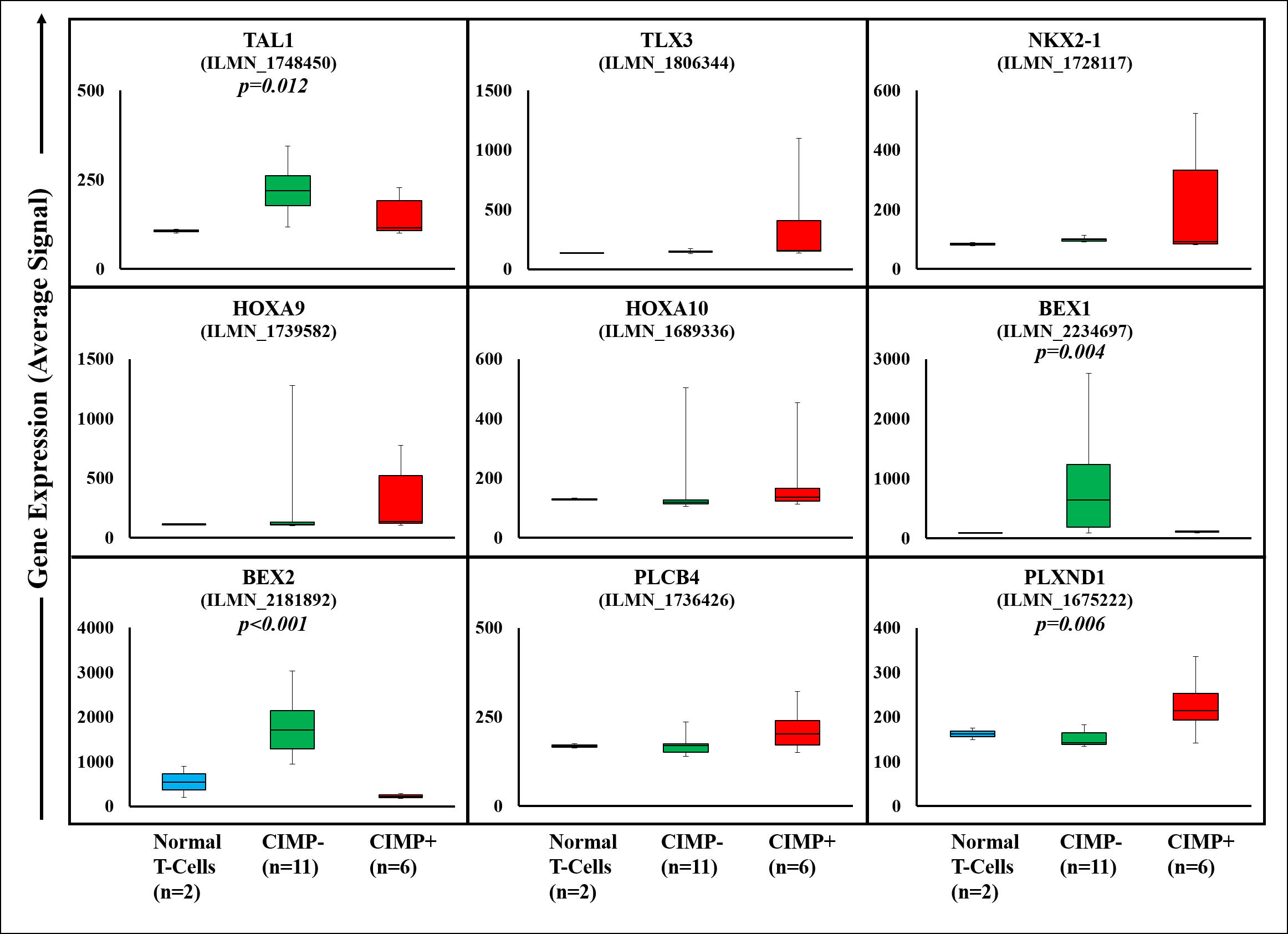

Supplement: Supplementary file 6 [file CAM4-8-311-s006.tif]
