# Supplementary material for: An integrated transcriptome analysis in T‐cell acute lymphoblastic leukemia links DNA methylation subgroups to dysregulated TAL1 and ANTP homeobox gene expression
Source: Cancer Med. 2018 Dec 21;8(1):311–24. doi: 10.1002/cam4.1917 (PMC6346238; doi:10.1002/cam4.1917)
Supplement: Supplementary file 10 [file CAM4-8-311-s010.pdf]

SUPPLEMENTARY FILE

TABLE OF CONTENTS

Supplementary Materials and Methods…………………………......................................2

Supplementary Tables…………………………………………………………….….9

Supplementary Table 1: Publicly available datasets used in the study ……................9

Supplementary Table 2: Epigenetic associated genes selected for analysis……….....10

Supplementary Table 3: Differentially expressed genes between CIMP subgroups...11

Supplementary Table 4: Enrichment pathway analysis……………………………...12

Supplementary Table 5: Variations detected in epigenetic associated genes by exome sequencing……………………………………...13

Supplementary Table 6: Gene Set Enrichment Analysis (GSEA) of DEGs…………14

Supplementary Table 7: Fusions detected by FusionCatcher………………………..15

Supplementary Figure Legends..…………....………………………………………….....16

SUPPLEMENTARY MATERIAL AND METHODS

Pre-processing and normalization of methylation array data

Pre-processing, normalization, and CIMP classification was performed as previously described by Borssen *et al*[1]. Briefly, CpG sites were removed 1) that had detection p-value <0.05, 2) that were located on the X or Y chromosomes, 3) where probe sequences did not uniquely align to the CpG locus, or 4) that were ≤3 base pairs away from a known single nucleotide polymorphism. The beta values of the different bead types in the HumMeth450K array was adjusted by BMIQ normalization[2]. The methylation value of each CpG site was given as an average β (avg. β) value ranging from 0 (completely unmethylated) to 1 (completely methylated).

Differential methylation analysis

The overall promoter methylation level for each sample was calculated by taking the average methylation of the CpGs, mapping to TSS1500 and TSS200 of all genes (n= 19 298) covered by the methylation array after filtering.

We used publicly available methylation data from sorted CD34+ cells from healthy individuals (NCBI GEO database GSE49618)[3] as a reference to investigate *de novo* methylation alterations in T-ALL. CpG sites were defined as hypermethylated or hypomethylated if they had a delta β value (Avg. β T-ALL sample - Avg. β CD34+ cells) of more than 0.4 or less than -0.4 respectively.

The enrichment of hyper- and hypomethylated CpG sites in the genomic and CpG island regions was calculated for each sample, as the ratio between the proportion of hyper/hypomethylated CpG sites within a region and the corresponding proportion in the HumMeth450K array.

Differentially methylated CpG sites (DM-CpGs) between the CIMP subgroups were defined as CpG sites with delta β value (mean methylation of CIMP+ subgroup – mean methylation of CIMP- subgroup) more than 0.4 or less than -0.4.

Copy-number variation analysis using methylation arrays

Total intensities of the methylation array probes were imported to R v.3.4.0 (R Core Team) using the minfi[4]. The genetic aberrations were identified from the total intensities using the conumee package[5] with two remission samples as reference group. Parameters for segmentation and limits for calling gain or loss were set individually for each sample by visual inspection. Regions including the DM-CpGs were identified and the number of samples with gains or deletions in the DM-CpG regions, was summarized for CIMP subgroups.

Telomere length measurement

For the relative telomere length (RTL) measurement, DNA samples were measured in triplicates in 96-well plates on the 7900 HT instrument (Applied Biosystems, Foster City, CA, US), and a mean RTL value was calculated for each sample. Mean RTL was calculated as previously described[6] with minor modifications[7]. Briefly, Ct-values (Ct_{TEL} and Ct_{HBB}) were obtained by quantitative real time PCR using telomere-specific primers and primers specific for a single-copy gene (HBB). T/S values were calculated for each sample by the T/S = 2−ΔCt method, where $\Delta Ct = Ct_{TEL}\text{-}Ct_{HBB}$. The RTL value for each sample were generated by dividing the sample T/S value with the T/S value obtained from the reference cell line (CCRF-CEM) which was included in all runs.

RNA-sequencing analysis

Sequencing libraries were constructed from a minimum of 600 ng RNA using the TruSeq stranded Total RNA library preparation kit with RiboZero Gold treatment (Illumina). The

libraries were subjected to paired-end sequencing on HiSeq2500 (Illumina) generating 125bp long reads.

The RNA sequence reads were analysed using the bcbio-nextgen pipeline v.1.0.1a0-664c8c6 (https://github.com/chapmanb/bcbio-nextgen). Data processing included quality control by FastQC v.0.11.5 (https://www.bioinformatics.babraham.ac.uk/projects/fastqc/), adapter trimming by Cutadapt v.1.12[8] and alignment to the reference genome (GRCh37/hg19) using the STAR aligner[9].

Reads were mapped to 57905 genomic features and raw counts were aggregated using HTSeq v.0.6.1[10]. The features were annotated by the Bioconductor package, biomaRt v.2.32.0[11] and the features annotated as “protein coding” (18692 features) were extracted. For the differential gene expression analysis between the CIMP subgroups, the filtering strategy outlined in Figure 2A was used.

The counts matrix of protein coding genes with mean counts >5 across the samples (13674 genes) were transformed using *regularized* log transformation (rlog) in the R Bioconductor package DESeq2 (version 1.16.1)[12]. The counts were transformed using the default settings of *rlog* with the “blind equals TRUE” argument. This ensured that the transformation of the samples was unbiased and didn´t depend on the CIMP status of the samples. The transformed counts were used to obtain Euclidean sample-to-sample distances to identify possible batch effects or confounding factors in the data. Additionally, principal component analysis (PCA) was used for exploration of potential batch effects using SIMCA v.14 (Umetrics, Umeå, Sweden). We concluded that varying RNA Quality Number (RQN) values, obtained using the Fragment Analyzer (Advanced Analytical Technologies) did not affect mapping statistics or introduce technical biases.

The DESeq2 package (version 1.16.1)[12] was further used for differential expression analysis using CIMP low samples as the reference. Genes with a false discovery rate (FDR) below 10%

(as per the default settings of DESeq2) and with an absolute $\log_2$ fold change (LFC) >1 or <-1 were defined as differentially expressed. Functional analysis was carried out in GeneGO MetaCore v.6.30 (Thomson Reuters, New York, USA).

In the validating gene expression analysis, gene expression data from Illumina HT-12 array of a separate Nordic T-ALL cohort (n=17) and stimulated T cells (n=2) (NCBI GEO database GSE42080)[13] was used (Supplementary Table 1).

Fusion detection by RNA-sequencing

FusionCatcher (version 0.99.6a)[14] was used to identify novel and known fusion transcripts with default parameters. The results were filtered to reduce false positives according to the strategy outlined in Figure 4A. Translocations annotated as "banned" or "healthy" were removed followed by removal of fusions involving ribosomal genes, small RNA, and possible read-throughs. Fusions supported by more than four paired reads spanning the junction were retained. STIL-TAL1 translocations were analysed manually on the junction track of Integrative Genomics Viewer (IGV) (versions 2.3.67)[15] using BAM files and hg19 genome sequence as reference. Sashimi plots[14] were subsequently generated also using IGV (Figure 4C).

Targeted exome sequencing

For targeted sequencing, 200 ng of genomic DNA was fragmented in eight mixes of restriction enzymes. Fragmentation was assessed by 2100 Bioanalyzer (Agilent Technologies, Palo Alto, CA, US) and the samples were hybridized to probes for three hours at 54°C following an initial ten minutes denaturation step. The circularized DNA-probe hybrids were next captured on streptavidin beads, washed and nicks in the probe- target hybrids were ligated. The captured target DNA was subsequently amplified in 50 µl reactions for 18 cycles and purified using AMPure XP beads (Beckman Coulter, Brea, CA, US). The enrichment was validated using

2100 Bioanalyzer and samples were pooled and sequenced on a MiSeq instrument (Illumina) using 2x151 base pair read sequencing.

The sequenced reads were analysed for somatic variants using bcbio-nextgen (version 1.0.6) cancer variant calling pipeline. Briefly, after quality control of the FASTQ files by FastQC (version 0.11.5), the reads were aligned to the reference genome (hg19) using Burrows-Wheeler Aligner (BWA) followed by variant calling using a variety of tools including FreeBayes (version 1.1.0-46-g8d2b3a0-dirty), GATK (version 3.7)[16] and samtools (version 1.6)[17]. The expected variant effects were then annotated using Ensembl Variant Effect Predictor (VEP) (version 83)[18] that included PolyPhen scores to predict the variant impact.

The identified variants were filtered according to the strategy outlined in Supplementary Figure 2A. Briefly, variants that were present in remission samples, in intronic or untranslated regions, or reported in the dbSNP[19] or 1000 genomes database (phase 3)[20] with an allele frequency of more than 1%, were filtered out. Variants occurring in regions with less than 40 reads across all samples were also filtered out. A total of 338 non-synonymous missense, frameshift, stop-gained, splice-donor or in-frame insertion/deletions were identified in 65 T-ALL samples after filtering. Out of these, 43 variants had an alternate allele frequency >20%.

Statistical analysis

Independent samples T-test and Mann-Whitney U test were used to compare differences between two subgroups for continuous variables. Two-sided Pearson correlation test was used to determine significance of linear correlations. All statistical tests for two sample hypotheses were two sided and were considered statistically significant if the p-value (p) was <0.05.

The normality assumption was assessed using Shapiro Wilks test. As the summary statistic in Table 1, mean is stated where the normality was not rejected whereas median was stated otherwise. However, for the enrichment scores, Mann-Whitney U test was used throughout for

consistency. The distribution of the means of chronological age (Table 1) in the subgroups can be assumed normal even though the data was not normally distributed as tested by normality tests. The assumption was made by manually inspecting age data which was approximately normally distributed but consisted of a small range of discrete values containing many ties. The statistical significance of gene expression differences between CIMP subgroups and normal T-cells (Supplementary Figure 6) was determined on log transformed values by using one-way ANOVA or the Kruskal-Wallis test where the normality assumption was not met. Homoscedasticity was assessed using Bartlett's (normality) or Levene's test which was rejected in BEX2 (Supplementary Figure 6), due to the large variation in the normal samples.

For comparing mitotic age and mean methylation in Figure 1, one-way ANOVA was used when the test of homogeneity of variances (Levene statistic) was not significant, and if significant, Welch´s one-way ANOVA was used.

For survival analysis, estimates of 3-year relapse free survival rates were calculated using the Kaplan-Meier method and the subgroups were compared using Mantel-Cox test.

Supplementary References

1. Borssen M, Haider Z, Landfors M, Noren-Nystrom U, Schmiegelow K, Asberg AE, et al. DNA Methylation Adds Prognostic Value to Minimal Residual Disease Status in Pediatric T-Cell Acute Lymphoblastic Leukemia. Pediatric blood & cancer. 2016;63(7):1185-92.
2. Teschendorff AE, Marabita F, Lechner M, Bartlett T, Tegner J, Gomez-Cabrero D, et al. A beta-mixture quantile normalization method for correcting probe design bias in Illumina Infinium 450 k DNA methylation data. Bioinformatics. 2013;29(2):189-96.
3. Cancer Genome Atlas Research N, Ley TJ, Miller C, Ding L, Raphael BJ, Mungall AJ, et al. Genomic and epigenomic landscapes of adult de novo acute myeloid leukemia. N Engl J Med. 2013;368(22):2059-74.
4. Aryee MJ, Jaffe AE, Corrada-Bravo H, Ladd-Acosta C, Feinberg AP, Hansen KD, et al. Minfi: a flexible and comprehensive Bioconductor package for the analysis of Infinium DNA methylation microarrays. Bioinformatics. 2014;30(10):1363-9.
5. Hovestadt VZ, M. . conumee: Enhanced copy-number variation analysis using Illumina DNA methylation arrays. R package version 1.9.0.

6. Cawthon RM. Telomere measurement by quantitative PCR. Nucleic Acids Res. 2002;30(10):e47.
7. Degerman S, Domellof M, Landfors M, Linder J, Lundin M, Haraldsson S, et al. Long leukocyte telomere length at diagnosis is a risk factor for dementia progression in idiopathic parkinsonism. PLoS One. 2014;9(12):e113387.
8. Martin M. Cutadapt removes adapter sequences from high-throughput sequencing reads. 2011. 2011;17(1).
9. Dobin A, Davis CA, Schlesinger F, Drenkow J, Zaleski C, Jha S, et al. STAR: ultrafast universal RNA-seq aligner. Bioinformatics. 2013;29(1):15-21.
10. Anders S, Pyl PT, Huber W. HTSeq--a Python framework to work with high-throughput sequencing data. Bioinformatics. 2015;31(2):166-9.
11. Durinck S, Spellman PT, Birney E, Huber W. Mapping identifiers for the integration of genomic datasets with the R/Bioconductor package biomaRt. Nat Protoc. 2009;4(8):1184-91.
12. Love MI, Huber W, Anders S. Moderated estimation of fold change and dispersion for RNA-seq data with DESeq2. Genome Biol. 2014;15(12):550.
13. Borssén M, Palmqvist L, Karrman K, Abrahamsson J, Behrendtz M, Heldrup J, et al. Promoter DNA methylation pattern identifies prognostic subgroups in childhood T-cell acute lymphoblastic leukemia. PLoS One. 2013;8(6):e65373.
14. Nicorici D, Satalan M, Edgren H, Kangaspeska S, Murumagi A, Kallioniemi O, et al. FusionCatcher - a tool for finding somatic fusion genes in paired-end RNA-sequencing data. bioRxiv. 2014.
15. Thorvaldsdottir H, Robinson JT, Mesirov JP. Integrative Genomics Viewer (IGV): high-performance genomics data visualization and exploration. Brief Bioinform. 2013;14(2):178-92.
16. McKenna A, Hanna M, Banks E, Sivachenko A, Cibulskis K, Kernytsky A, et al. The Genome Analysis Toolkit: a MapReduce framework for analyzing next-generation DNA sequencing data. Genome Res. 2010;20(9):1297-303.
17. Li H, Handsaker B, Wysoker A, Fennell T, Ruan J, Homer N, et al. The Sequence Alignment/Map format and SAMtools. Bioinformatics. 2009;25(16):2078-9.
18. McLaren W, Gil L, Hunt SE, Riat HS, Ritchie GR, Thormann A, et al. The Ensembl Variant Effect Predictor. Genome Biol. 2016;17(1):122.
19. Sherry ST, Ward MH, Kholodov M, Baker J, Phan L, Smigielski EM, et al. dbSNP: the NCBI database of genetic variation. Nucleic Acids Res. 2001;29(1):308-11.
20. Genomes Project C, Auton A, Brooks LD, Durbin RM, Garrison EP, Kang HM, et al. A global reference for human genetic variation. Nature. 2015;526(7571):68-74.
21. Pongers-Willemse MJ, Seriu T, Stolz F, d'Aniello E, Gameiro P, Pisa P, et al. Primers and protocols for standardized detection of minimal residual disease in acute lymphoblastic leukemia using immunoglobulin and T cell receptor gene rearrangements and TAL1 deletions as PCR targets: report of the BIOMED-1 CONCERTED ACTION: investigation of minimal residual disease in acute leukemia. Leukemia. 1999;13(1):110-8.

SUPPLEMENTARY TABLES

Supplementary Table 1: Publicly available datasets used in the study

**Sample Type**	**Number of Samples**	**Data Type**	**Platform**	**NCBI GEO Dataset Accession Number**	**Reference**
Sorted CD3+ T-cells and CD34+ cells from healthy donors (Bone Marrow)	6	Methylation	HumMeth 450K Array	GSE49618	*Cancer Genome Atlas Research et al., 2013*
Peripheral blood samples of healthy children	78	Methylation	HumMeth 450K Array	GSE36064	*Alisch et al., 2012*
Pediatric T-ALL	17	Gene Expression	Illumina HT12	GSE42080	*Borssén et al., 2013*
Normal Stimulated T-cells	2	Gene Expression	Illumina HT12	GSE42080	*Borssén et al., 2013*
Pediatric T-ALL	65	Methylation	HumMeth 450K Array	GSE69954	*Borssén et al., 2016*

Supplementary Table 2: Epigenetic associated genes selected for analysis

TargetID	Description	Targeted exome sequencing	RNA sequencing
BMI1	Polycomb group (PcG) protein	×	×
CBX2	Polycomb group (PcG) protein	×	×
CBX4	Polycomb group (PcG) protein	×	×
CBX6	Polycomb group (PcG) protein	×	×
CBX7	Polycomb group (PcG) protein	×	×
CBX8	Polycomb group (PcG) protein	×	×
DNMT1	Methyltransferase	×	×
DNMT3A	Methyltransferase	×	×
DNMT3B	Methyltransferase	×	×
EED	Polycomb group (PcG) protein	×	×
EZH1	Polycomb group (PcG) protein	×	×
EZH2	Polycomb group (PcG) protein	×	×
JARID2	Polycomb group (PcG) protein	×	×
MTF2	Polycomb group (PcG) protein	×	×
PCGF1	Polycomb group (PcG) protein	×	×
PCGF2	Polycomb group (PcG) protein	×	
PCGF3	Polycomb group (PcG) protein	×	×
PCGF5	Polycomb group (PcG) protein	×	×
PCGF6	Polycomb group (PcG) protein	×	×
PHC1	Polycomb group (PcG) protein	×	×
PHC2	Polycomb group (PcG) protein	×	×
PHC3	Polycomb group (PcG) protein	×	×
PHF1	Polycomb group (PcG) protein	×	×
PHF19	Polycomb group (PcG) protein	×	×
RBBP4	Chromatin Modifier	×	×
RBBP7	Chromatin Modifier	×	×
RING1	Polycomb group (PcG) protein	×	×
RNF2	Polycomb group (PcG) protein	×	×
RYBP	Chromatin Modifier	×	×
SCML1	Polycomb group (PcG) protein	×	×
SCML2	Polycomb group (PcG) protein	×	×
SUZ12	Polycomb group (PcG) protein	×	×
YAF2	Chromatin Modifier	×	×

Supplementary Table 3: Differentially expressed genes between CIMP subgroups

Supplied as an additional excel file

Supplementary Table 4: Enrichment pathway analysis of differentially expressed genes.

**Cluster A**				
#	**Maps**	**Total**	**p-value**	**FDR**
1	Development_Transcription regulation of granulocyte development	32	2.12E-04	3.98E-02
2	Signal transduction_mTORC2 downstream signaling	68	4.23E-04	5.29E-02
3	Immune response_CRTH2 signaling in Th2 cells	44	7.35E-04	6.40E-02
4	Development_Role of G-CSF in hematopoietic stem cell mobilization	21	9.36E-04	6.40E-02
5	Immune response_NF-AT signaling and leukocyte interactions	48	1.02E-03	6.40E-02
**Cluster B**				
#	**Maps**	**Total**	**p-value**	**FDR**
1	Breast cancer (general schema)	41	2.02E-04	4.63E-02
2	Transport_ACM3 in salivary glands	42	2.31E-04	4.63E-02
3	G-protein signaling_Regulation of Cyclic AMP levels by ACM	45	3.41E-04	4.63E-02
4	Development_G-protein-mediated regulation of MAPK-ERK signaling	46	3.85E-04	4.63E-02
5	Signal transduction_Activation of PKC via G-Protein coupled receptor	52	7.53E-04	6.48E-02

Supplementary Table 5: Variations detected in epigenetic associated genes by exome sequencing.

Supplied as an additional excel file.

Supplementary Table 6: Gene Set Enrichment Analysis (GSEA) of DEGs.

GSEA of higher expressed genes in CIMP- (LFC<-1) (Cluster A)						
**NAME**	**SIZE**	**ES**	**NES**	**NOM p-val**	**FDR q-val**	**RANK AT MAX**
CLUSTER_2	13	-0.75	-3.17	0.00	0.00	203
CLUSTER_3	5	-0.84	-2.25	0.00	0.00	125
CLUSTER_4	2	-0.64	-1.15	0.27	0.55	276
CLUSTER_0	1	-0.76	-1.02	0.44	0.66	185
CLUSTER_5	1	-0.72	-0.97	0.54	0.61	213
CLUSTER_12	1	-0.68	-0.91	0.63	0.59	243
**GSEA of higher expressed genes in CIMP+ (LFC>1) (Cluster B)**						
CLUSTER_11	8	0.62	1.91	0.01	0.01	172
CLUSTER_8	9	0.56	1.77	0.02	0.02	216
CLUSTER_9	5	0.62	1.56	0.04	0.05	193
CLUSTER_10	15	0.34	1.33	0.15	0.14	467
CLUSTER_7	2	0.66	1.15	0.28	0.28	20

Column Description:

Name-Cluster name (Souleir et al 2005)

Size-Number of common genes in the respective clusters and differentially expressed gene list

ES-Enrichment Score

NES-Normalized Enrichment Score

NOM p-val-Nominal p value

FDR p-val-False Discovery Rate

Supplementary Table 7: Fusions detected by FusionCatcher

Supplied as an additional excel file.

SUPPLEMENTARY FIGURE LEGENDS

Supplementary Figure 1. Correlation of copy-number variations and methylation levels at differentially methylated CpGs (DM-CpGs) between the CIMP subgroups.

The DM-CpG regions (n=12 063), identified between the CIMP subgroups (Figure 1B), were grouped on the x-axis based on the presence and/or absence of deletions and gains, and classified further by CIMP subgrouping (CIMP- green boxes, CIMP+ red boxes) of the samples they occur in. The y-axis represents the mean methylation level of the DM-CpG in the respective genetic aberration group (none/deletion/gains/both).

Supplementary Figure 2. Differential *de novo* methylation alterations in pediatric T-ALL.

De novo **A)** hyper- and **B)** hypomethylated CpG sites (delta β >0.4 or delta β <-0.4, respectively) in 65 T-ALL samples using normal CD34+ cells as a reference. Pearson coefficient (R^2) of the correlation between the number of hyper- and hypomethylated CpG sites and CIMP methylation percentage is given. Enrichment of hypermethylated CpG sites in **C)** gene centric and **D)** CpG island regions is compared between the CIMP subgroups. *** p <0.001 (Mann-Whitney U test)

Supplementary Figure 3: Targeted exome sequencing of epigenetic regulators.

A) The filtering strategy for the variations identified by targeted exome sequencing. The variations were annotated by the Ensembl Variant Effect Predictor (VEP) and filtered for true positives. Number of variations retained after each filtering step is stated. **B)** The final variants in 11 genes, retained after filtering, in the 65 T-ALL diagnostic samples (25 CIMP- and 40 CIMP+) sorted by increasing CIMP methylation percentage. Samples marked with (*) were RNA-sequenced (n=30).

Supplementary Figure 4: Expression profile of known epigenetic regulators in T-ALL.

The heatmap shows gene expression (rlog counts) of known epigenetic regulators and enzymes in 30 diagnostic T-ALL samples, sorted by increasing CIMP methylation percentage.

Supplementary Figure 5: STIL-TAL1 fusion validation by PCR.

A) Amplification of genomic DNA using STIL-TAL1 primers specific for TAL1 breakpoint 1 (taldb1)[21] in 64 T-ALL samples. The CIMP- samples (n=24) are labeled in green and the CIMP+ samples (n=40) are labeled in red. Samples marked with (*) were RNA-sequenced. The CCRF-CEM T-cell line was used as a positive control for the fusion and the negative control had no DNA template. **B)** The amplification of sample X70 that had a different TAL1 breakpoint (TAL1 deletion breakpoint 7, taldb7), using taldb7 specific primers.

Supplementary Figure 6: Expression analysis of selected DEGs by Illumina arrays.

Illumina HT-12 gene expression array levels (average signal) of pediatric T-ALL samples (n=17) and normal stimulated T-cells grown in vitro (n=2) analyzed in the study by Borssen *et al*. 2013[11]. The groups are compared either by one-way ANOVA test or by the non-parametric Kruskal-Wallis test and the significant p-values ($p < 0.05$) are stated.
